# A qualitative exploration of the impact of educational social fields on mental health help‐seeking in post‐primary schools in Northern Ireland

**DOI:** 10.1111/1467-9566.13825

**Published:** 2024-07-29

**Authors:** Bethany Waterhouse‐Bradley, Dagmar Corry, Gerard Leavey

**Affiliations:** ^1^ The Bamford Centre for Mental Health & Wellbeing Ulster University Belfast UK; ^2^ School of Psychology Queens University Belfast Belfast UK

**Keywords:** education, help‐seeking, mental health, social class, trust

## Abstract

In a study of post‐primary students in Northern Ireland, Bourdieu’s concepts of cultural capital, habitus and fields are used to explore attitudes towards help‐seeking from general practitioners (GPs). Findings from Grammar and Secondary Modern School students are compared using the role of educational fields in influencing help‐seeking behaviours for mental health problems. Focus groups were conducted of 54 students at 10 post‐primary schools in Northern Ireland, each consisting of 5–7 pupils, stratified by age (13–17 years) and gender. The data were analysed thematically to assess attitudinal and belief patterns within school environments. Participants from both types of schools expressed reluctance to seek help from GPs for mental health concerns. However, the attitudes towards help seeking differ between grammar schools (GSs) and secondary modern schools with regards to (a) the act of help‐seeking; (b) service knowledge and medical professionalism; and (c) trust and disclosure. The field of GSs appears to produce students who feel more able and, importantly, more entitled to mental health support from health professionals. While this apparent conference of cultural norms increases some individuals’ access to services, work is required to build help‐seeking pathways which are responsive to diverse young people.

## INTRODUCTION

Adolescence is a transitional period of life from puberty to adulthood, usually accompanied by efforts towards identity construction. During this stage, young people strive to separate themselves from their parents, but lack a clearly defined role in society. While increasing numbers of young people experience poor mental health (Gore et al., 2011; Meltzer et al., 2003), only one third of those with a diagnosable mental illness ever get professional help (Green et al., 2005; World Federation for Mental Health, 2009), even for severe problems (Biddle et al., 2006; Goodman et al., 2002). The reluctance of young people to consult for mental health problems blocks early intervention (Rickwood et al., 2007; Rothi & Leavey, 2006) and is costly for the individual, families and society. The decision to seek support for mental health problems is heavily determined by social factors—particularly the socially internalised perceptions of health professionals and treatment options (Billings & Young, 2021).

Social class has been identified as a potentially pivotal factor in determining access to services and various health outcomes (Marmot et al., 1991). In the context of persistent evidence of a social gradient in the prevalence of mental illness, Holman (2014) points to the underrepresentation of working‐class people in obtaining ‘talking therapies’ and evidence of higher rates of prescription medication. Such disparities may be explained by structural factors relevant to help‐seeking (e.g. costs, service availability and access, discrimination) and power resources and dynamics related to class, the challenges of illness appraisal, articulation and presentation among working class individuals towards middle‐class professionals. Cultural factors (health beliefs and service preferences) can also shape perceptions of entitlement or usefulness (Bourdieu & Wacquant, 1992). Given the emphasis on trust and presentation in successful health seeking, some disparities can be linked to family milieux and cultural backgrounds which may influence children’s help‐seeking attitudes and behaviours related to emotional distress and mental health problems (Leavey et al., 2020). As such, Bourdieu’s social reproduction theory has relevance to understanding these differences. Cultural and social capital can be used to understand attitudes towards and competencies in help‐seeking. It can also elucidate reasons behind some mental health decision‐making (cultural capital), and the issue of trust between (and among) young people and adults, parents, and professionals (social capital). Cultural capital itself consists of the social norms, relational behaviours, grammar and vernacular and cultural and aesthetic preferences, which are conferred upon or absorbed through social networks and connections and familial social and institutional contexts in which individuals are embedded (Bourdieu, 1997). Cultural capital is conceptualised in three primary ways: objectified (objects or assets which are recognised by dominant group members are having cultural significance), institutionalised (awards and credentials within the system) and embodied (linguistic skills, reading music) (Dumais, 2001). Cultural capital as a form of power and advantage has been linked positively to improved health outcomes. This has been deemed to be linked to embodied cultural capital, often referred to in more individualistic public health messaging as ‘lifestyle choices’, like healthier food and activities. These inequalities can also be linked to institutional capital, such as the nature and quality of relationships and interactions with professionals. These are often directly related to the way elite actors in the system prioritise certain cultural values over others (Whitehead et al., 2016). Shim (2010) dubbed this ‘cultural health capital’.

Social capital, the benefits generated through ‘contacts and group memberships, through the accumulation of exchanges, obligations and shared identities, provide actual or potential support and access to valued resources’ (Bourdieu, 1993, p. 143) which are also relevant. These resources translate into somewhat obvious benefits. For example, children from professional or middle‐class backgrounds are more likely to have contact with relatives and family friends from health professional and educational fields. These relationships offer advantages through extended networks of knowledge, expertise and signposting—and an increased capacity to understand and navigate entrenched hierarchical institutions. These associations have the potential to generate trust in other members of those groups, and therefore increase the likelihood of being willing to seek support from similar professionals in future. There is also scope for these pathways to facilitate an increased sense of coherence and higher control beliefs, which have shown to have positive impacts on health outcomes (Whitehead et al., 2016). These advantages intersect with other non‐health choices and can often be linked to the habitus, or the embodied practices and grammar of everyday life which individuals inherit and develop through their social connections and environments.

Habitus (Bourdieu, 1985) offers a framework to aid understanding of class related health behaviour, including health care access. Part of that understanding is informed by cultural attitudes to mental illness and coping responses to adversity and emotional distress, and different levels of emotional literacy or knowledge about psychological therapies. Habitus suggests that social groups differentially approach the world with a set of dispositions and practices that are maintained and developed, albeit partially, in response to their own economic position. Cultural differences, whether ethnic or social class related, may influence concepts and behaviours related to gender and identity, stigma, autonomy, stoicism and endurance and coping mechanisms. Lareau discusses the difference between working class and middle‐class parents in willingness to assert their needs and wishes with authority figures, and the likelihood of them passing this on to their own children (Lareau, 2003). As Lareau (2015) suggests, middle‐class children have a better understanding of the ‘rules of the game’. Inherent in all of this is the fact that ‘the game’ is controlled by those with socioeconomic advantage, and thus the ‘rules’ are determined in accordance with those norms. This embeds disadvantage for those who do not conform to established social rules, resulting in persistent health inequalities which have frequently been connected to day‐to‐day interactions with those who wield professional power to confer appropriate support (Holman, 2014). Additionally, the collective experience of socioeconomic disadvantage has the scope to lead to a lower sense of agency and control over one’s own experiences on an individual level, and a collective sense of mistrust or powerlessness which impacts mental health and the sense of being able to address associated problems (Whitehead et al., 2016).

Contrary to criticism of habitus being too deterministic a concept, Bourdieu (1990) explains the habitus of an individual to be relatively fluid and to be shaped as environmental expectations and the individual aspirations fluctuate (Edgerton & Roberts, 2014). The context in which this is formed, shaped and practiced is referred to as the field. Habitus developed through one field (family, community) can and will be shaped and re‐shaped by other fields (e.g. educational environments). As such, while a grammar school (GS) is not an exact indication of higher socioeconomic class, it is certainly indicative of a field which values and rewards the habitus of higher socioeconomic class and where there is scope for habitus to be transformed. Given the impact of these components on the cultural capital, and the cognitive choices which can and are likely to be shaped by this, a GS provides a reasonable proxy for social class. This setting also gives insight into the impact of educational field on the transformation of habitus through conferring help‐seeking behaviours and skills, shifting attitudes towards mental health and the determining the level of trust allocated to health‐care professionals. Also important to this is the relational dynamics between health‐care professionals, a group dominated by higher socioeconomic classes and associated behaviours/language (Holman, 2015), and those help‐seekers from lower socioeconomic backgrounds. Systematic approaches to the transfer of cultural capital in the form of help‐seeking behaviours are shown to be effective in improved academic outcomes for students (Richards, 2020).

### Grammar and secondary modern schools as culture shaping fields

Help‐seeking is characterised as a set of values and orientations, specialised information, strategies and sets of skills. The latter of these can be cultivated through proactive efforts to obtain and/or transfer skills, and the educational field—even the secondary school environment specifically—has been found to be a place where the cultural asset of help‐seeking can be transmitted (Richards, 2020). There is evidence that social fields are potential sites for the acquisition of help‐seeking as cultural capital beyond the family. This change in help‐seeking disposition is the result of the educational field as a mechanism for habitus transformation (Ibid.). Schools have been identified as places where an imbalance of cultural capital can be mitigated. While this is often discussed in the context of educational outcomes, attitudes towards help‐seeking and mental health and trust in professionals all constitute aspects of social and cultural capital, which could also be improved through the educational field. Relationships between authority figures and pupils, the way mental health is discussed and the language used are all affected by the social field and as such provide opportunities for intervention to support students in utilising health professionals for mental health services.

Nash (2005) draws on ethnographic data to support the idea that the educated habitus required to achieve entry to a GS is underpinned by key non‐cognitive concepts such as self‐discipline, self‐control and confidence of communication. GSs have long been a point of critical discussion about social class and educational attainment. Thus, entrance to such schools is selective based on an entrance exam at aged 11. High‐attaining students from lower socioeconomic backgrounds in England are still significantly less likely to attend a GS than their peers from higher socioeconomic backgrounds. Looking at similar statistics in England, those from the 10th Socioeconomic status (SES) centile only 6% attended a GS, as opposed to 51% of students from the 90th centile, increasing to 79% of the top 1%. Half of all GS places in England are occupied by students from families belonging to the highest 25% income group (Burgess et al., 2018).

While most of the UK moved away from the process of academic selection, Northern Ireland has retained the practice and continues to educate children at grammar and secondary schools. According to the 2020/2021 Schools Census, GSs comprise 43% of all post‐primary enrolments in Northern Ireland, compared with approximately 5% in England (Denichi, 2020). Despite the large proportion of the student population in GSs, 14% of GS students in Northern Ireland received free school meals (FSMs) as opposed to 40% in secondary schools (Gallagher, 2019). Because of the prevalence of GS in Northern Ireland, this jurisdiction provides us with more insight into the habitus transforming potential of the educational field as the schools may be largely populated by one socioeconomic class or another, they are still sites for social mobility given their relative accessibility when compared with, for example, fees paying schools.

Using the ‘structure—disposition—practice explanatory scheme (SDP)’, Nash (2005) warns that the use of proxies in some quantitative research often ignores the lived experiences which underpin habitus while focusing on the objectified cultural capital and undervaluing the practice‐based habitus and context of the field. The use of GSs as a proxy for social class in this study places the emphasis firmly *on* the structural implications of the field environment (the grammar vs. secondary school) and considers the habitus fostered and integrated through successfully navigating that field.

According to Bourdieu (1986), capital, habitus and field are interdependent in social reproduction. We hypothesised that GSs and secondary modern schools would act as fields of practice, which are largely linked to and follow norms of differences between social classes in help‐seeking behaviours. GS students are more likely to exhibit practices linked to help‐seeking behaviours, attitudes towards mental health decision‐making and the social norms of trust of authority in higher SES. This advantages them in the process of help‐seeking in environments which value such norms (Manstead, 2018). This is likely because of the predominance of students and educators from higher SES present in the field, as well as the likelihood that those students from lower socioeconomic backgrounds who have successfully navigated GS are more likely to embody practices, both cognitive and non‐cognitive, which are in line with the predominant expectations (Nash, 2005). We sought to explore the attitudes and beliefs of young people in seeking help from general practitioners (GPs), and how these are influenced by GS as a field which influences habitus and cultural capital of its pupils.

In a longitudinal, ethnographic study within a socioeconomically diverse public elementary school, Calarco (2011), in the USA, showed that social class influences how and when children seek help in the classroom. Compared to their working‐class peers, middle‐class children request more help from teachers and employed different strategies. They were more direct and assertive in their approach and consequently received more attention and help from teachers. Calarco observed that the use of these skills and strategies contributes to inequalities by building advantages for middle‐class children in the classroom. It seems plausible that the confidence and skills required to obtain professional help, and the advantages of this level of educational, social and cultural capital are extended into other spheres such as familial ability to recognise mental health problems and their knowledge of different psychological therapies (educational capital).

## METHODS

This study was part of a larger research programme which sought to examine the prevalence of factors associated with mental health problems in adolescents and to examine help‐seeking behaviours of adolescents. The study had a particular focus on the role of the GP (Leavey et al., 2011, 2020). General practice is a key site for understanding trust and help‐seeking among adolescent because the family doctor is commonly the first point of contact within formal health‐care services and the interface where young people are expected to develop a sense of agency in the management of health, independent of parents and guardians. This mixed methods study involved a cross‐sectional survey, data linkage and focus groups with students from the same schools which undertook the survey. Findings from the quantitative study supported the qualitative design, including development of the topic guide and coding frame (Leavey et al., 2020).

### Wider study design

The wider research included a cross‐sectional survey undertaken by pupils across Northern Ireland between the ages of 13–17 years. The analysis included linked data from school administrative records which allowed for examination of factors such as area deprivation codes, FSMs, academic performance and school attendance. The survey included measures of mental health, trust GPs, willingness to seek help from GPs, home life, family life, family affluence and the Warwick and Edinburgh mental wellbeing scale. Informed consent was obtained by parents and students, and students completed the questionnaire in the school setting, utilising methods undertaken in a previous study (Leavey et al., 2020). *Ethical approval* for the full study was granted by the Ulster University Research Ethics Committee. Respondents from the quantitative study provided the sampling frame for the focus groups, though their unique responses were not linked to their participation in the qualitative study.

### Sample

Participants were selected from the schools involved in the wider research study. Eight schools were selected from an education and library board database and stratified by type and deprivation indices supplied by the Northern Ireland Statistical Research Agency. In Northern Ireland, children aged 11 years sit an ‘entrance’ exam to determine whether they go to a GS or a ‘secondary modern’ school (SMS). All pupils were invited to take part in the study, and experience of mental health problems was not an inclusion requirement. While data on socioeconomic background in the focus groups was not collected, the pool from which these students were drawn was similar to that of the wider study (survey sample), in which students at secondary modern schools were almost three times as likely to be eligible for FSMs.

Twenty‐four girls and 30 boys between the ages of 13 and 16 attending eight post‐primary schools in Northern Ireland participated in nine focus groups, each consisting of between five and seven pupils. The groups were organised by age (13–17) and gender (limited to male and female identifying), and students took part in groups comprised entirely of students from their own school. GSs made up half of the sample of post‐primary schools.

### Focus groups

On the basis of findings from the survey (Leavey et al, 2020), previous research (Leavey et al, 2020) and the help‐seeking and mental health literature, we constructed a topic guide covering the following areas for discussion: (a) familiarity with family doctor, (b) the roles of the family doctor for emotional and mental health, (c) barriers to contacting GP about emotional or mental health issues and (d) help‐seeking preferences. Focus groups lasted approximately 60 min and were facilitated by one member of the research team. The sessions were digitally audio‐recorded, transcribed and entered into NVivo 10.

### Analysis

Three researchers assisted in the analysis, coding in vivo for emergent themes. The researcher doing the initial coding for the present study did not take part in the data collection process, and as such was doing a blind analysis. The researcher was not initially told which schools were grammar and which were secondary modern while doing the first codes. Regular meetings were held to discuss the coding, analysis and conclusions.

Using a classic analysis strategy best exemplified by Miles and Huberman (1994) we worked towards a broad thematic description of help‐seeking strategies of the participants and then developed a more finely grained exploration of beliefs and attitudes related to professional help‐seeking and mental illness. These have been described in a previous paper (Corry & Leavey, 2016). Through initial data analysis, there was an apparent divergence in attitudes towards help‐seeking and trust in professionals in some focus groups. This led to further exploration of these themes for the purposes of this article. Again, the initial coding was done in vivo and by a researcher who was not involved in the data collection, and the researcher was blinded to the type of school while going through the focus group data. Here, we sought to explore data which indicated the habitus (including cognitive and non‐cognitive practices) of pupils regarding help‐seeking, attitudes towards professionals, perceptions and expectations of mental health support and the relationship between those attitudes and behaviours and the educational field (e.g. references to relationships to authority figures, social norms in the school environment).

**Code**: I (interviewer); R (respondent)

## FINDINGS

Young people, regardless of social class, highlighted the stigma attached to mental illness and the difficulties of seeking help. However, participants from grammar schools (GS) and secondary modern (SMS) schools show different perceptions of (a) help‐seeking from adults; (b) service knowledge and medical professionalism and (c) trust and disclosure. Finally, there are several findings which indicate a relationship between a shared cultural and social capital in respective schools, the acceptability/appropriateness of seeking help for mental health problems, and the willingness to utilise GP services to that end. In most cases, the students were able to give specific examples or point to experiences with authority figures, carers or support providers which reinforced these perspectives. The consistency of responses across students within each respective school environment. The connection between attitudes and environmental/relational experiences gives weight to the likelihood that these attitudes relate to similar social class norms and were shaped by the GS and SMS fields.

### Stigma

Stigma towards poor mental health and help‐seeking is strongly linked to the social norms of groups. GS participants argued that the treatment of mental health problems within primary care ought to be given the same consideration as any other health problem—an emphasis on the mundanity and thus ‘normality’ of such problems. This kind of acceptance and parity of esteem (with other problems) would create an environment which facilitated a greater willingness to seek help. They emphasised the entitlement of the individual to a level of care and consideration based on their need, and there was an expectation that professionals should be expected to provide treatment without judgement.

As if it was another normal condition—not that it’s different or strange—just that it’s another normal thing and to be treated the same as everyone else.

(Male, GS)

I’d like him to treat me like a normal person, not like “I think there’s something wrong with him.”

(Male, GS)

Not always see you as a depressed person. Like if somebody gets sick or has a stomach bug, they don’t always see them as a person that’s sick but if you’re depressed, they might just see you as a depressed person but rather than that just seeing it as a normal condition.

(Male, GS)

I feel like maybe—I wouldn’t go to try to get advice for things but one of the biggest things I think in depression would be if you think no‐one else is going through it, it’s only me but if you were to go on the internet and there were lots of other stories, you would know it’s not as rare as you think it is and that would make you want to go to the GP and think it’s ok to go there.

(Male, GS)

Participants from SMS variously reported experiences of ‘getting into trouble’ or being admonished for feeling depressed or anxious. SMS participants showed patterns of mental‐health attitudes which were more focused on extrinsic or collective concerns—how they would be perceived by the group or how their mental health might affect others.

R If you talked to them about suicide or something like that then they’d feel like they were going to shout at you or get annoyed at you or tell you basically to wise up.

I So, you might think that maybe sometimes they might not take it seriously?

R They would treat you differently as well, I reckon, with suicide or something like that, completely differently.

R They would lose all their trust in you as well. They wouldn’t trust you to go outside.

R Like you wouldn’t be left in your room, you wouldn’t have a minute to yourself, they’d be like constantly on your case, making sure you are alright and asking are you ok or do you want anything. (Male respondents, SMS)

Notable in these differences is the adoption of the medicalised framing of mental health from GS students, indicating an internalisation of a wider shift towards labelling and formalising mental health problems in neoliberal institutions. On the contrary, SMS students equated medical intervention as being necessary only for the abnormal, and in extreme circumstances. They had little expectation of having their needs met, predicting either an overreaction or no reaction at all. Gender also came into play more significantly when considering stigma, with the emphasis on normality or medicalisation being more prominent from male respondents across both groups.

### Social trust and relationships to authority figures

SMS students, and more frequently, male SMS students, reported reluctance to go to professionals at the risk of being doubted, shamed and/or pitied. Boys, from SMS schools expressed assumptions that they would not be taken seriously by teachers, or that their confidence would not be respected.

R1 See if you told our year Head about our problems, he’d just tell you to wise up.

R2 Aye he just goes—if you go to him and talk, he’s just like “get the violin out”.

(Male respondents, SMS, when asked about discussing problems with other trusted adults)

Because he’s just not a person you would trust—he could go off into the staff room and tell everyone and show the whole school in assembly.

(Male, SMS, talking about whether he would use a school counsellor)

Trust no one…everyone has a big mouth.

(Male, SMS)

SMS boys recalled teachers accusing them of faking or exaggerating, trying to get out of class, or being weak or disruptive if they said they needed to talk to someone about mental health issues.

When I’m sick he says “you’re lying” and how’s he supposed to know? He’s not in my mind, he’s not feeling what I’m feeling.

(Male, SMS, referring to a teacher in the school)

He’d [teacher] just say “why you want to go for”—and then I would be like “I’m not telling you” and he’d be like, “you just want out of class”—that’s what he’d say.

(Male, SMS)

Some respondents took this a step further, articulating a concern that they would be penalized or problematized for their disclosure.

If you went to like your Head and said “Can I go to the drug counsellor”, he’d be like “what are you taking drugs for?” and then he’ll go straight to your mum or dad.

(Male, SMS)

This level of fear of antagonism and distrust tends to be generalised and is often based on previous experiences with authority figures. Thus, SMS participants were wary of anyone with institutionalised power. They also tended to equate administrative processes and formalities with these figures. For example, one group of boys spoke at length about their suspicion when people wrote things down or kept written records of their previous visits. This was particularly the case with personal details.

R1 They take your number and your address and that’s why I don’t feel confidential in there.

R2 Yeah so if they’re taking your number and your emergency contact, then they’re obviously not confidential if they do that…

R1 We don’t know if they would pass it on or not.

R2 Because they still had all files from the last time I was there.

(Male respondents, SMS, discussing visiting a clinic)

This reluctance extended into recording things themselves, either through emails or online forums. Many SMS students showed a reluctance to put anything in a format that could be saved. In one discussion, it was noted that ‘internet’ evidence could later be used against them (see issues with fearing punitive measures if they ‘confess’ mental health issues). Here we see both a lack of expectation from authority figures (cultural capital) and low levels of trust in professionals—both of which appear to have been reinforced by the school environment in SMS experiences.

### Service knowledge and professionalism

The cultural skill of help‐seeking was one which appeared to be poorly supported and reinforced by authority figures in SMS in a manner which is consistent with the expectations of medical professionals, whereas GS students appeared to have more knowledge and understanding of what are considered appropriate processes and networks for seeking help in medical communities and professional settings. GS students appeared to have better awareness of the roles of specific service providers and how to access them. Additionally, GS students gave more weight to professionals generally, which affected their willingness to approach or trust them. This translated into differences of interpretation of specific behaviours—even where both behaviours were deemed to be negative. The previously stated example of note‐taking denotes this difference. There was a general suspicion of professional note‐taking amongst both groups; however, among GS participants, this was highlighted as an issue of professional distance and objectification—indicating a lack of personal connection, rather than anxiety about potential conflict with institutional power.

We noted that concern with confidentiality and institutional power is contingent, with considerably different views expressed about professional health services other than GPs. Thus, despite of expressing concern over how many personal details recorded at a sexual health clinic, one group of boys regarded these as trustworthy places where confidentiality was guaranteed. He highlighted the legal ramifications for the clinic about disclosure, indicating a level of faith in legal sanctions as a deterrent for unprofessional behaviour.

It’s like a sex clinic or else you can talk about your emotional problems…You get tested for stuff and you get condoms and all or else you can go and talk about problems and stuff…Yep, they tell you that if they give out information that they get put in jail.

(Male, SMS, when asked who could be trusted to talk to)

GS participants were more informed about the GP’s role than pupils from the SMSs. Additionally, they appeared more knowledgeable about how to access services and showed a greater respect for scientific professionalism, confidentiality and training; notably remarking on limitations of parental capacity to provide help for mental health problems. Their positive regard for medical staff stands in contrast to that of the SMS pupils who commonly expressed uncertainty, scepticism and distrust of their GPs.

R1 They [GP] explain it in a really medical scientific way –.

R2 Yeah.

R3 whereas your parents explain it in a really down to earth way.

(Both Female, SMS)

If you’re sick and self‐harming, your parents are just going to [tell you] don’t self‐harm. The GP is going to tell you what you should do.

(Male, GS)

Yeah, I would feel fine with it because I know [GPs are] offering proper advice—they are trained in what they do and it stays all confidential and stuff so I feel like it would be ok to talk to them.

(Male, GS)

I think tell a doctor because your parents wouldn’t—doctors have to be professional—your parents just don’t know really.

(Female, GS)

Interestingly, while the GS participants highlighted the detached professionalism of GPs (compared to parental understanding), the secondary modern school students tended to regard this less positively, as indicative of having no compassion or concern.

R1 Your mum and dad may understand you but your GP they are just hearing the same story every day, so they don’t really care.

R2 They sort of just have to listen to you because it’s their job.

R3 It’s their job to listen to you.

(Male respondents, SMS)

### Trust and disclosure

Participants, regardless of school type, portrayed an anxiety that GPs may lack sincerity in dealing with young people’s emotional and mental health problems. For example, they felt that their family doctor understood the rudiments of help‐seeking and had a ‘script’ for what they were ‘supposed’ to say. Young people complained that they had little faith that they would be ‘listened to’ and helped. Again, that the GP had no personal interest in the patient.

I don’t know—if they were showing that they really could help you—that there was something they could actually do rather than just you telling them and then finding out that you just have to wait again—that there was something meaningful in the meantime.

(Male, GS)

And if they were listening, you know that they are going to remember you; Doctors I think just—it kind of looks like they are pretending to listen and they really only pick out the key points and they try to read it but you think that you’ve kind of stuck out in this person’s mind because the way you’ve worded and your whole background of it.

(Female, SMS)

However, overall, the SMS students tended to describe their experiences with GPs or other health professionals as negative. Again, there was a stronger sense among the SMS discussions of being ‘uncared for’ or disregarded within primary care. Perhaps as a corollary, they also talked more about being offered medication by the doctor than GS students. Medication was seen as a substitute for proper concern and understanding, or professional advice and counselling.

R1 The way it’s normally only ten minutes per patient, they kind of push you away sometimes.

R2 Yeah you feel like you’re running out of time.

R3 They’re rushing you.

R2 Yeah.

R3 When really things like those matters should be taken slowly, I think.

(Female respondents, SMS)

R1 Like some of them just give you medicine. Then they just forget about you—you don’t know when …

R2 They move on to the next patient.

(Female respondents, SMS)

They mightn’t help you, they might just prescribe something and say what’s your problem and then just tell you to get out—not actually tell you—you know, kind of rush you out like without actually telling you how to help yourself without taking tablets or whatever.

(Female, SMS)

Because obviously that [checking to see how you’re feeling] wouldn’t be their first thing in their mind—“Oh well I have to go check on up this or her.” It won’t be the first thing on their mind because you would just be like—“how they do on the medicine?”.

(Female, SMS, explaining why she thought GPs were less sympathetic than counsellors)

These negative experiences overall are linked to a pronounced sense of disconnection and distrust of GPs. In the following extracts from the secondary modern school pupils’ discussions these sentiments ripple across the group responses and can be tied directly to the lack of continuity of care at their surgeries.

R1 Every time I’ve been to the doctors I got a different one, so.

R2 It’s the same with me.

R3 I don’t even know what a GP is.

R2 I’d feel uncomfortable.

R4 Yeah, I’d feel uncomfortable.

R5 I wouldn’t—I wouldn’t talk to the GP because I don’t think I could trust them.

(Male respondents, SMS)

Yeah there’s like a trust barrier cos you don’t know if you can trust them or if they’re going to keep it confidential or if they’re going to respect you.

(Female, SMS)

When asked how a GP could assist in developing a more trusting relationship, SMS participants emphasised intimacy building behaviours. They expressed desire for reciprocity, feeling cared for, and knowing personal details about the practitioner to trust them. This suggestion seemed to be related to extracting family doctors from their institutional contexts and their authority role and creating an egalitarian level of intimacy through mutual knowledge.

He could tell you something—like a secret about him, I don’t know…He could tell you like something private about him.

(Male, SMS)

Feel like your friend that doesn’t just talk to you when you have a problem—like chat to him. Not just talk to him when you are—talk to him like text him or ring him or something.

(Male, SMS)

GS Students discussed the possibility of seeing a GP, stating clearly what they would need from a GP to seek help from them. In contrast, SMS participants were much less willing to consult a GP, even when directly asked what a GP might do to be more accessible.

Yeah, you wouldn’t really feel comfortable telling them like—Or I wouldn’t anyway but then if you had to tell them you would—but I wouldn’t go out of my way to tell them.

(Female, SMS)

SMS groups did not always dismiss the idea of being able to eventually build trust with a GP altogether, but the requirements for building that trust were more rigorous; some requiring regular, even daily visits over an extended period to build familiarity and trust. This emerged from a perceived need to test the relationship and extract evidence of investment. Interestingly, participants emphasised the importance of direct face‐to‐face contact while signifying the superficiality of digital messaging contact. These were evident in the responses given to the question ‘how could you feel the GP was interested enough for you to trust them’.

R1 Cancel other appointments if it’s going to drag on more and she wants to help me more.

R2 They’ll say, “well I’ve got another appointment, but I’ll cancel that and I’ll stay with you”.

R3 Do some overtime just to help people, voluntary.

(Male respondents, SMS)

R1 Someone who you could talk to daily, that you trust one hundred percent and that you knew that he or she wouldn’t say anything … I wouldn’t talk over text message or anything because that means they just have the text –

R2 It would be face to face.

R1 I’d rather it be face to face.

(Male respondents, SMS)

Yeah, like somebody you could go to. I know we have counsellors and stuff like that but somebody you could go to maybe every day of the week instead of if something happened one day instead of just having to be waiting for them to be able to talk to you or something like that.

(Female, SMS)

This need for intensive contact was also noticeable in some GS students but was more related to professional clinical monitoring and as proof that they cared, rather than building initial trust pre‐disclosure.

Trust towards GPs was commonly acknowledged across groups as developed through familiarity—over time and with a high level of direct personal contact. Even so, GS students often knew a GP personally or described having a direct link to the health service through family or close social networks. Thus, GS participants described greater continuity with their GP and were aware of their right to see a specific GP. They were confident to ask to see their preferred provider and were prepared to wait for an appointment.

This more consumer‐based attitude translated into GS students indicating greater confidence in the ability of the GP to know what he/she was doing regarding treatment. Even when they said they would not like to go to a GP over a counsellor, many conceded that the GP might be more likely to know what to do. Moreover, the GS pupils were able to provide a more nuanced discussion about professional continuity, trust‐building and service needs, indicating that their more positive and familiar experiences have given them space to reflect on rather than react to the relationship.

R1 It wouldn’t be the first thing you would think of. Like normally your parents or a helpline but maybe if you see that they could help, maybe they would be helpful if you did.

R2 I was aware that you can go and see your GP if there’s something wrong.

(Male respondents, GS)

Because if you’re talking to a GP online you’re still not as exposed but at the same time still getting professional advice rather than just hearing people’s stories on forums.

(Male, GS, on the prospect of talking to a GP online about mental health problems)

I’d like if it was either (a) somebody who you already know and trust; or (b) somebody who you don’t really know at all; but somebody in the middle, who you know but you aren’t particularly comfortable around would be quite bad.

(Female, GS)

They understand the more—the smaller points about your condition—they might notice things that if you were going to a different doctor every time, they mightn’t notice but if you’re going to the same doctor, they might see new things developing or the finer points about it.

(Male, GS, when asked about why he asks to see the same doctor every time)

Those SMS students who reported feeling comfortable with a GP stated that this was due to a long‐established relationship with an individual GP, rather than trusting GPs more generally.

Because usually you’re very close like with my doctor, I’ve known him since I was a baby so I’ve just been going to the same doctor so it’s much easier to talk to them and maybe my parents as well.

(Female, SMS)

Maybe if it’s your family doctor for a few years, you might know and you might tell him.

(Male, SMS)

GS participants, more so than SMS participants, acknowledged something of the wider mental health system, discussing the possibility of a GP referral to specialist mental health professionals, noted as a potential drawback to seeking help from a GP. Thus, they discussed their preference for consultation with a specialist mental health centre for young people.

Somebody who specifically—I don’t know—maybe GPs are trying to deal with this but maybe somebody who’s specifically trained to do deal with this, they might be able to deal with it in a better way and as you said, if you go to your GP and you have to be referred, there’s long waiting times and something could happen to make your condition worse and that wouldn’t be very good.

(Female, GS)

That’s the thing, like if you could set up a specified centre for mental health or whatever, they would be dealing with this and they would know that you need the time to talk or is it the GP—it’s everything and they have to fit everyone in.

(Male, GS)

## DISCUSSION

There is evidence that fields can impact help‐seeking disposition (Abraham et al., 2017; Richards, 2020), as well as shaping the levels of trust and communication in educational and health‐care settings (Leavey et al., 2011). While GSs cannot be a direct proxy for social class, as educational environments which have strong traditional foundations in class norms and which continue to have an over‐representation of pupils and staff from higher socioeconomic backgrounds, GSs as fields have the capacity to significantly impact or reinforce the habitus in a manner which affects the help‐seeking propensity of the students. Moreover, there were observable distinctions and differences in help‐seeking attitudes and behaviour between these groups. This does allow for some inference which can be helpful in complementing literature exploring the role of social class in the educational field. If one’s power to act is limited by the influence and pressures of the field in which one is practicing, it is important to consider the interdependent relationship of habitus and the field.

Our findings suggest that there are shared anxieties among young people as they consider their relationships with GPs, such as wanting to be listened to and respected rather than patronised. Commonly, they feel that GPs lack sincerity in dealing with young people’s emotional and mental health problems. Fears about the stigma of mental illness and the need for confidentiality resonate across all groups. The differences become apparent, however, when examining the attitudes towards mental health through normative attitudes towards mental wellbeing, and expectations of how they should and will be treated by professionals. This related to the cultural capital of help‐seeking as a social and cognitive skill (Nash, 2005), as well as the social capital of trust in professionals.

These findings showed pupils from GS were more likely to see medicalisation of mental health as favourable, and importantly, core to the normalisation of these issues. These pupils had high expectations of professionals, and a sense of entitlement to confidential consistent care. GS fields appear to be more effective at conferring strong expectations about how young people should be treated by authorities, and a better knowledge of the skills and roles of service providers and professionals. Expectations of professionalism and intimacy from health professionals appear to be influenced by their experiences with authority figures within the educational environment, in the health‐care field and in their home environments. Exposure to individuals in positions of authority who treat the students with a sense of agency and respect are likely to increase trust (social capital) and transform habitus regarding self‐perceived competence in relating to professionals and authority figures. GS students were much more likely to relay these types of experiences, and to reflect on their own rights and preferences when considering help‐seeking because of this conferred agency. GS students were more likely to have reported personal connections with GPs, consistency in their treatments and accessibility of services. GS pupils mirrored typically middle‐class expectations of care, attitudes towards professionalisation and a favouring of the medicalised model of mental wellbeing. This is likely indicative of home environment and the educational field, where these students are practicing interactions using the acceptable scripts and tools of that environment. The communicative and social tools internalised and practiced in the GS are more likely to be deemed appropriate in other hierarchical environments, such as medicine, given the predominance of pupils from higher income backgrounds (Edgerton & Roberts, 2014). This is consistent with Whitehead et al.’s (2016) theoretical synthesis, which identified patterns of higher control beliefs where communities were collectively empowered.

This is contrasted with SMS pupils, who were likely to express scepticism and mistrust around professionals, and a resistance to labelling or medicalising mental health problems. They believed seeking help for mental health problems would lead to being accused of lying or exaggerating or elicit disproportionate reactions, including punishment. These young people were more likely to believe their confidence would be broken by professionals, and that personal information was more likely to be used against them than to help them. While trust from GS students was likely to be granted based on professional qualifications alone, SMS pupils sought more intimate and reciprocal means of negotiating this relationship. Contrary to this, SMS children tended to anticipate negative and critical responses from significant adult others. The stories they used to articulate these reservations often related to their experiences with teachers and professionals within their school and these are generalised to help‐seeking from their doctors. While on the surface the reported discomfort with recording information and mistrust of authorities could be perceived as excessive, there is evidence that the day‐to‐day experience of these young people underpins these beliefs. Students from lower socioeconomic backgrounds are more likely to experience surveillance, be detained against their will and be criminalised for their behaviour (Eubanks, 2018). The demand for relationship building and reciprocal trust makes sense when one considers middle‐class therapists are less likely to identify with working class patients and find it difficult to build rapport (Holman, 2014). They are more likely to pathologise their behaviours, and to misinterpret familial situations and cultures to the detriment of their patients (Coutant & Eideliman, 2013).

It is also worth acknowledging that while there was a mix of perceived genders in the groups, there was limited ethnic diversity, and participants were not asked to disclose or discuss other aspects of their identity (e.g., beyond the male/female gender binary, disability, sexual orientation). Research exploring the intersection of these identities would enhance future research in this area. Significantly, it might be argued that we have adopted an overly ‘realist’ approach in our interpretation and presentation of the interview data and that group discussions may not truly reflect actual behaviours in real‐life scenarios. This is a reasonable argument and merits further consideration.

Different educational fields yield students with different approaches to seeking mental health support. The interpretation of these findings, however, cannot be extracted from the social and political dynamics in which these fields operate, and by which they are shaped. Health outcome discrepancies based on socioeconomic inequality can often be attributed to physical deprivation but is also often linked to institutional barriers and sociocultural factors. This indicates the importance of better understanding the fields of practice and cultural capital which can positively and negatively impact health outcomes. Health inequalities are directly related to an imbalance of power (Marmot et al., 1991), and power differentials related to socioeconomic inequality are evident in the context of GS and SMS in Northern Ireland. This was supported by the findings of this study.

## CONCLUSIONS

While improved mental health literacy correlates highly with educational attainment and leads to higher likelihood of diagnosis and treatment for mental health (Holman, 2015), it is important not to fixate on accessibility of mental health services without due consideration to the appropriateness and responsiveness of these treatments to heterogeneous groups (Holman, 2014). There is evidence that situated within a neoliberalised hierarchical health system, talking therapies can act as another form of control of young people, shifting responsibility for support from the state to the individual and family networks (Coutant & Eideliman, 2013). Studies of adolescent help‐seeking need to consider the complexity that social class adds to our understanding of trust and contact with health professionals, and to recognise the importance of fields of practice in perpetuating behaviours and beliefs which can reinforce inequalities in health. Further, these issues need to be incorporated into a more granulated approach to school programmes and interventions for better mental health. While the creation of habitus through the educational field can be a protective factor for some young people, it clearly indicates societal barriers for help‐seeking that require reform of health services.

## AUTHOR CONTRIBUTIONS

**Bethany Waterhouse‐Bradley**: Conceptualization (supporting); data curation (equal); formal analysis (equal); funding acquisition (supporting); investigation (supporting); methodology (equal); project administration (supporting); resources (supporting); software (equal); supervision (supporting); validation (equal); visualization (equal); writing—original draft (equal); writing—review and editing (lead). **Dagmar Corry**: Formal analysis (supporting); methodology (supporting); project administration (supporting); writing—original draft (supporting); writing—review and editing (supporting). **Gerard Leavey**: Conceptualization (lead); formal analysis (supporting); funding acquisition (lead); methodology (lead); project administration (lead); resources (lead); software (equal); supervision (lead); validation (equal); visualization (equal); writing—original draft (equal); writing—review and editing (equal).

## Data Availability

The data that support the findings of this study are available on request from the corresponding author. The data are not publicly available due to privacy or ethical restrictions.
